# Expression of Genes Encoding Manganese Peroxidase and Laccase of *Ganoderma boninense* in Response to Nitrogen Sources, Hydrogen Peroxide and Phytohormones

**DOI:** 10.3390/genes11111263

**Published:** 2020-10-26

**Authors:** Pei-Yin Ho, Parameswari Namasivayam, Shamala Sundram, Chai-Ling Ho

**Affiliations:** 1 Department of Cell and Molecular Biology, Faculty of Biotechnology and Biomolecular Sciences, Universiti Putra Malaysia (UPM), Serdang 43400, Selangor, Malaysia; heatherhappy91@gmail.com (P.-Y.H.); parameswari@upm.edu.my (P.N.); 2 Ganoderma and Diseases Research of Oil Palm, Malaysian Oil Palm Board, Bandar Baru Bangi, Kajang 43000, Selangor, Malaysia; shamala@mpob.gov.my; 3 Institute of Plantation Studies, Universiti Putra Malaysia (UPM), Serdang 43400, Selangor, Malaysia

**Keywords:** *Ganoderma*, hydrogen peroxide, laccase, manganese peroxidase, nitrogen, phytohormones

## Abstract

*Ganoderma* produces lignolytic enzymes that can degrade the lignin component of plant cell walls, causing basal stem rot to oil palms. Nitrogen sources may affect plant tolerance to root pathogens while hydrogen peroxide (H_2_O_2_), salicylic acid (SA) and jasmonic acid (JA) play important roles in plant defense against pathogens. In this study, we examined the expression of genes encoding manganese peroxidase (MnP) and laccase (Lac) in *Ganoderma boninense* treated with different nitrogen sources (ammonium nitrate, ammonium sulphate, sodium nitrate and potassium nitrate), JA, SA and H_2_O_2_. Transcripts encoding MnP and Lac were cloned from *G. boninense*. Of the three GbMnP genes, GbMnP_U6011 was up-regulated by all nitrogen sources examined and H_2_O_2_ but was down-regulated by JA. The expression of GbMnP_U87 was only up-regulated by JA while *GbMnP_35959* was up-regulated by ammonium nitrate but suppressed by sodium nitrate and down-regulated by H_2_O_2_. Among the three *GbLac* genes examined, *GbLac_U90667* was up-regulated by ammonium nitrate, JA, SA and H_2_O_2_; *GbLac_U36023* was up-regulated by JA and H_2_O_2_ while *GbLac_U30636* was up-regulated by SA but suppressed by ammonium sulphate, sodium nitrate, JA and H_2_O_2_. Differential expression of these genes may be required by their different functional roles in *G. boninense*.

## 1. Introduction

Palm oil production is threatened by a fungal disease known as basal stem rot (BSR) which is mainly caused by *Ganoderma boninense*. Infected oil palms may not show obvious symptoms at the initial stage of infection. The foliar symptoms expressed by infected oil palms include unopened spear leaves, yellowing leaves, small canopy and flattened crown. The lower stem rotted at the advanced stage of BSR with the emergence of basiodiocarps [1].

*Ganoderma* species are white rot fungi which can degrade lignin by producing extracellular ligninolytic enzymes including manganese peroxidase (MnPs; EC 1.11.1.13) and laccase (Lac; EC 1.10.3.2) [2,3]. MnP oxidizes Mn^2+^ to Mn^3+^ and nonphenolic aromatic compounds with high oxidation-reduction potentials such as lignin [4]. Lac, which is catalyzed by the redox ability of copper ions, can also oxidize nonphenolic substrates with high oxidation-reduction potentials, concomitantly with the reduction of oxygen to water [5,6,7]. Purified and well characterized Lac enzymes from different *Ganoderma* strains have been reported in a few studies [8,9,10]. Molecular cloning of genes encoding MnP [11] and Lac from *Ganoderma lucidum* [12,13] and their heterologous expression have also been reported [11,14]. Nevertheless, little is known about the expression of genes encoding MnP and Lac and their enzyme activities in *G. boninense* which causes BSR in oil palms except that the genes encoding MnPs and Lacs from *G. boninense* were reported to be up-regulated in infected oil palms [15].

The production of lignolytic enzymes from white-rot basidiomycetes was found to be suppressed upon application of nitrogen fertilizer [16]. Extremely low nitrogen content was reported as the primary cause for the extensive delignification while high nitrogen concentrations inhibit lignin degradation [17]. Nitrogen-starved fungus may gain access to nitrogen in the lignoprotein complex that forms a major portion of nitrogen in the wood [18]. *G. lucidum* grown in high-nitrogen (24 mM) produced a higher Lac level in shaken cultures than that grown in low-nitrogen (2.4 mM). Low nitrogen (arginine) content also stimulated the production of MnP [19]. A high incidence of *Ganoderma* infection in oil palm plantations was reported when the soil nitrogen was high [20]. The application of a high level of urea also increased the percentage of *Ganoderma* incidence in oil palm [21]. However, the regulation of genes encoding MnP and Lac in *G. boninense* by nitrogen remains unknown.

Hydrogen peroxide (H_2_O_2_), salicylic acid (SA) and jasmonic acid (JA) play important roles in plant defense against pathogens [22]. SA- and JA-dependent defense responses are triggered in plant hosts against biotrophic and necrotrophic pathogens, respectively; while reactive oxygen species (including H_2_O_2_) are involved in both plant signaling and defense [22]. It was reported that phenolic compounds affect the secretion and activity of lignolytic enzymes in *G. boninense* [23]. However, the effects of SA, JA and H_2_O_2_ on the gene expression of MnP and Lac in *G. boninense* are not known. Nevertheless, SA was demonstrated to suppress the growth of *G. boninense* [24,25] while JA was reported to improve the growth of *G. boninense* [24]. In addition, SA was found to suppress basidiocarp formation on rubber wood [25], and reduce the BSR disease incidence in oil palms grown in greenhouses [26]. Although the catalytic activity of MnP is well known to be dependent on H_2_O_2_; the enzyme is also inactivated by excess H_2_O_2_ [27]. Low amounts of H_2_O_2_ (2.93 and 14.69 mM) stimulated MnP activity, whereas high amounts of H_2_O_2_ (29.39 and 293.99 mM) inhibited MnP activity in white rot fungi [28]. In addition, the white-rot fungus *Phanerochaete chrysosporium* was found to produce MnP mRNA following the addition of a low amount of H_2_O_2_ in the absence of Mn^2+^ [29]. On the other hand, H_2_O_2_ was also reported to interact with one specific Cu^2+^ binding to the Lac enzyme [30].

Since the BSR incidence in oil palm plantations was found to be affected by nitrogen levels [20,21] and SA [26], and SA, JA and H_2_O_2_ may be involved in oil palm defense against *Ganoderma* infection [15], we hypothesized that nitrogen sources, JA, SA and H_2_O_2_ could regulate the expression of lignolytic enzymes that are MnP and Lac that cause BSR in oil palm. However, the MnP and Lac enzyme activities in *G. boninense* could be contributed by a few enzyme isoforms in the MnP and Lac families; therefore, in this study, we cloned the transcripts encoding three MnP and three Lac enzymes from *G. boninense* (abbreviated as GbMnPs and GbLacs, respectively); and measured their gene expression in *G. boninense* PER71 treated with four different nitrogen sources (ammonium nitrate, ammonium sulphate, sodium nitrate and potassium nitrate), JA, SA and H_2_O_2_. These transcripts were chosen because they were found to be expressed in *G. boninense* that infected oil palms in a previous transcriptome study [15]. The findings of this study may shed light on the gene regulation of lignolytic enzymes that play an important role in lignin degradation of oil palms by *G. boninense.*

## 2. Materials and Methods 

### 2.1. Preparation of Fungal Cultures

Czapek–Dox broth (35 mL) supplemented with different nitrogen sources, in other words, 50 mM ammonium sulphate, ammonium nitrate, sodium nitrate and potassium nitrate, respectively, was inoculated with eight mycelial discs of *Ganoderma boninense PER71* in each 125 mL-conical flask. Untreated *G. boninense PER71* in Czapek–Dox broth was used as control for nitrogen treatments. Three replicates were prepared for each treatment and control. The flasks were kept in the dark as static cultures at 30 °C. The mycelia of *G. boninense* were harvested at 6 days post-inoculation (dpi). 

For phytohormone and H_2_O_2_ treatments, 35 mL potato dextrose broth (PDB) was prepared in each 125 mL-conical flask and inoculated with eight mycelial discs. The medium was supplemented with 1 mM salicylic acid (SA) (Sigma-Aldrich, St. Louis, MO, USA), or 1 mM jasmonic acid (JA) (Sigma-Aldrich, USA) or 1 mM H_2_O_2_. Untreated *G. boninense* in PDB was used as control. Three replicates were prepared for each treatment and control. All treatments were kept in the dark and incubated as static cultures at 30 °C for 4 days. The mycelia of *G. boninense* were harvested at 4 dpi. The amount of phytohormones was determined based on [24,25,26] while the amount of H_2_O_2_ was determined based on [28].

The mycelia were collected by filtration using Whatman filter paper No. 1, rinsed with distilled water to remove the remaining medium and dried by pressing on filter paper. The mycelia collected were frozen in liquid nitrogen and stored at −80 °C for RNA extraction.

### 2.2. Enzyme Assays

Secreted enzymes were collected from liquid cultures of *G. boninense* by centrifugation at 10,621 g for 30 min at 4 °C. Crude enzymes in the clear supernatants contained were used for enzyme assays. The activity of MnP (EC 1.11.1.13) was determined based on [31] in triplicate. The reaction mixture contained 0.5 M sodium succinate buffer (pH 4.0), 1 mM of manganese sulphate (MnSO_4_), 0.1 mM H_2_O_2_, 1 mM guaiacol (Acros Organics, Fair Lawn, NJ, USA) as enzyme substrate and 500 μL crude enzyme. Absorbance reading was measured at 465 nm. The molar absorption coefficient at 465 nm (E_465_) at 12.1 mM^−1^ cm^−1^ was used for the calculation of enzyme activity. Laccase (EC 1.10.3.2) enzyme activity was determined as described by [32] in triplicate. The assay mixture contained 1 mM 2,2’-azino-bis(3-ethylbenzothiazoline-6-sulphonic acid) diammonium salt (ABTS) (Sigma-Aldrich, USA), 100 mM sodium acetate buffer (pH 5.0) and 500 μL cell-free crude enzyme. Absorbance reading was measured at 420 nm. The molar absorption coefficient at 420 nm (E_420_), at 36.0 mM^−1^ cm^−1^ was used for the calculation of enzyme activity. One unit (U) of enzyme activity is defined as the amount of enzyme needed to transform 1 μmol of product in one minute under the assay conditions. Differences between the means were analyzed using analysis of variance (ANOVA). Significant differences among mean values (at *p* < 0.05) were estimated using Duncan’s multiple range test.

### 2.3. Inoculation of Oil Palms with G. boninense in the Presence of Different Nitrogen Sources

Rubber wood blocks (6 cm × 6 cm × 6 cm) colonized by *G. boninense* were used for artificial inoculation of three-month-old Dura x Pisifera GH500 oil palm seedlings (Sime Darby Seeds and Agricultural Services Sdn. Bhd., Banting, Malaysia). Treatment 1 consisted of uninfected oil palm seedlings treated with different nitrogen sources while Treatment 2 consisted of *G. boninense*-infected oil palm seedlings (infected by using sitting technique as described in [33]) treated with a once-off treatment of 250 mL of 50 mM ammonium sulphate, 50 mM ammonium nitrate, 50 mM sodium nitrate and 50 mM potassium nitrate at the beginning of the experiment. Three replicates were used for each treatment. Disease symptoms of oil palm seedlings were evaluated weekly based on a modified disease severity scores for 24 weeks as shown in Table S1. The disease severity index (DSI) was calculated as mentioned in [34], where DSI (%) = (∑(score frequency × disease severity scores))/((total number of plants) × (maximal disease severity scores)) × 100.

### 2.4. Primer Design from Partial Transcripts

The partial transcript sequences (unigenes) encoding MnP and Lac from a previous study [15] were analyzed by Basic Local Alignment Search Tool (http://blast.ncbi.nlm.nih.gov). Different sets of gene-specific primers (GSPs) for rapid amplification of cDNA ends (RACE) were designed and checked for specificity by using Primer3 (http://bioinfo.ut.ee/primer3-0.4.0/primer3/) and Oligonucleotide Properties Calculator (http://biotools.nubic.northwestern.edu/OligoCalc.html). The primer sequences are shown in Table S2.

### 2.5. Total RNA Extraction and RACE-PCR

Total RNA was extracted by using a cetyl trimethylammonium bromide (CTAB)-method [35] followed by treatment with DNase I (New England Biolabs, Hitchin, UK) according to the manufacturer’s instructions prior to cDNA synthesis. The first-strand cDNA was synthesized according to the protocol of SMART RACE cDNA amplification kit (Clontech, Mountain View, CA, USA). Amplification of the 5′, 3′, or both ends, of transcripts was conducted by using RACE-PCR according to the manual (Clontech, USA). The cycling conditions for the PCR program were 30 cycles of 94 °C for 30 s, 68 °C for 30 s and 72 °C for 3 min. The PCR products obtained were analyzed on 1.5% (*w*/*v*) Tris-acetate-EDTA (TAE) agarose gel followed by gel purification using MEGAquick-spin Plus Total Fragment DNA Purification Kit (iNtRON, Seongnam-Si, Korea). The RACE-PCR products with A-overhang were cloned into yT&A vector (Yeastern Biotech, New Taipei City, Taiwan). The ligated PCR products were transformed into 100 μL of *Escherichia coli* DH5α. The plasmid DNA was confirmed by colony PCR for restriction enzyme analysis before Sanger sequencing.

### 2.6. Sequence Analysis of Full-Length cDNA

The open reading frame (ORF) was predicted by using BioEdit Sequence Alignment Editor Version 9.0 [36]. The deduced protein sequences were aligned by using ClustalW in BioEdit Sequence Alignment Editor Version 9.0. The neighbor-joining tree was generated by using MEGA5 program with a bootstrap of 1000 replicates [37]. The signal peptides and cleavage site were predicted by using SignalP 5.0 (http://www.cbs.dtu.dk/services/SignalP/) [38]. The GenBank accession numbers are as follows: GbMnP_U87 (MT559133); GbMnP_U6011 (MT559134); GbMnP_U35959 (MT559135); GbLac_U30636 (MT559136); GbLac_U36023 (MT559137) and GbLac_U90667 (MT559138).

### 2.7. Quantitative Reverse Transcription-PCR (qRT-PCR)

Gene expression analysis was conducted for *G. boninense* samples from treatments with different nitrogen sources in Czapek–Dox broth for 6 days, and from treatments with JA, SA and H_2_O_2_ in PDB collected at 4 dpi; compared with respective control treatments. Affinity Script qPCR cDNA Synthesis Kit (Stratagene, La Jolla, CA, USA) was used to synthesize cDNA from DNase-treated RNA samples. Real time PCR was performed in iQ5 iCycler Thermal Cycler (Bio-Rad, Hercules, CA, USA) with Brilliant SYBR Green QPCR Master Mix (Stratagene, USA). The primers used are shown in Table S3. The relative transcript abundance of each target gene was normalized to that of housekeeping genes (α-tubulin and β-tubulin; [39]). Differential gene expression was considered as significant when relative abundance of transcript in treated *G. boninense* to that in untreated *G. boninense* in either Czapek–Dox broth (control for nitrogen treatments) or PDB (control for JA, SA and H_2_O_2_ treatments), was ≥2-fold (up-regulation) or ≤0.5-fold (down-regulation) [40].

## 3. Results and Discussion

### 3.1. Sequence Analysis of GbMnPs and GbLacs in G. boninense

**Figure 1:**
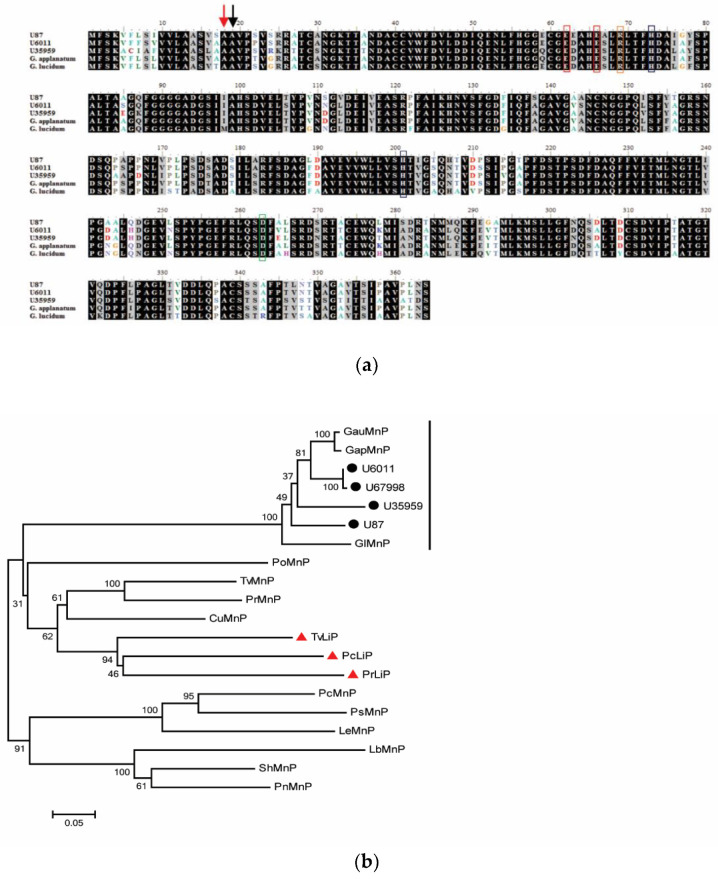
Sequence alignment and phylogenetic tree of GbMnPs. (**a**) Multiple sequence alignment of GbMnPs. The cleavage sites of predicted signal peptide for U87 (red), and U6011 and U35959 (black) are indicated with arrows. Distal and proximal histidine residues are indicated by blue rectangles. Red rectangles show the manganese binding sites. The orange rectangle indicates the distal arginine residue while the green rectangle indicates H-bonded aspartate residue. The conserved amino acid is shown with black background. The accession numbers for MnPs from *Ganoderma applanatum* (BAA88392.1) and *Ganoderma lucidum* (ACA48488.1) are in parentheses. (**b**) Neighbor-joining tree of deduced amino acid sequences of MnP from *G. boninense* PER 71 and other fungal MnPs. Numbers at the branches show bootstrap support of 1000 replicates. The tree is drawn to scale, with branch lengths having the same units to evolutionary distance denoted by the horizontal bar (0.05 amino acid substitution per site). MnP sequences obtained from this study are labeled with black circles and LiP sequences retrieved from NCBI are labeled by red triangles. Abbreviations of MnPs from: *Cerrena unicolor* (CuMnP, AGS19355.1); *Dichomitus squalens* LYAD-421 SS1 (DsMnP, EJF61830.1); *Ganoderma australe* (GauMnP, ABB77244.1); *Ganoderma applanatum* (GapMnP, BAA88392.1); *Ganoderma lucidum* (GlMnP, ACA48488.1); *Laccaria bicolor* S238N-H82 (LbMnP, XP_001888065.1); *Lentinula edodes* (LeMnP, BAG72079.1); *Phanerochaete chrysosporium* (PcMnP, AAA33743.1; PcLiP, AAA33739.1); *Phlebia radiata* (PrMnP, CAC84573.1; PrLiP, AAW71986.1); *Pholiota nameko* (PnMnP, BAU36966.1); *Pleurotus ostreatus* (PoMnP, AAA84396.1); *Punctularia strigosozonata* HHB-11173 SS5 (PsMnP, XP_007384391.1); *Stereum hirsutum* FP-91666 SS1 (ShMnP, EIM81487.1) and *Trametes versicolor* (TvMnP, AAD02880.1; TvLiP, AAA34049.1). The vertical bar indicates the *Ganoderma* MnPs.

**Figure 2:**
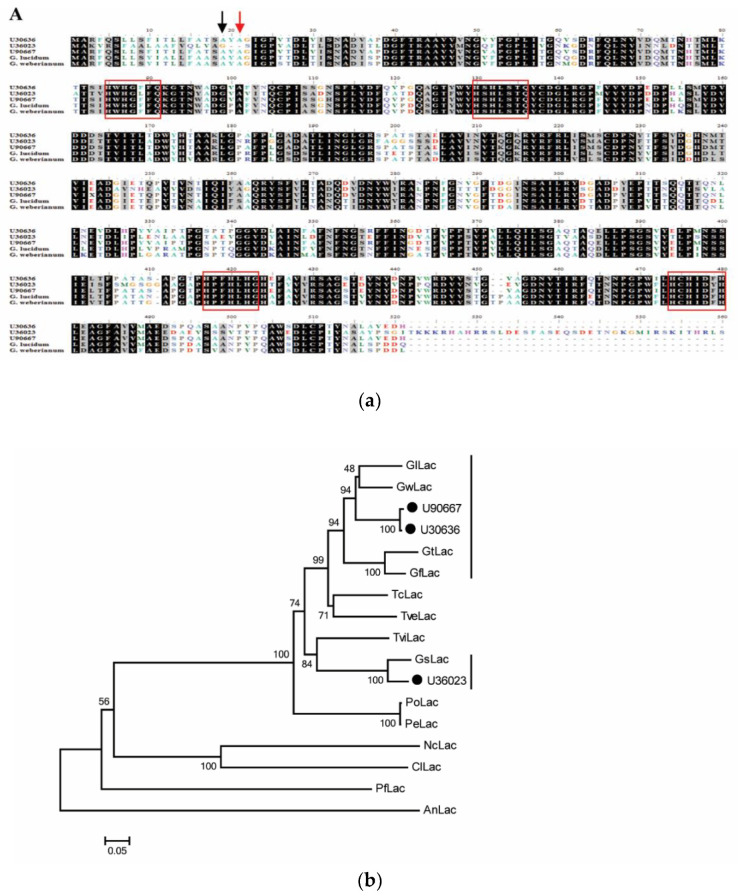
Sequence alignment and phylogenetic tree of GbLacs. (**a**) Multiple alignment of GbLac protein sequences from *G. boninense*, *G. lucidum* (Accession number: AHA83584.1) and *Ganoderma weberianum* (accession number: ANA53145.1). The cleavage sites of predicted signal peptide for U30636 and U90667 (red), and U36023 (black) are indicated with arrows. Red rectangles show copper-binding domains. The conserved amino acid is shown with black background. The accession numbers for Lacs from *G. lucidum* (ACR24357.1) and *G. weberianum* (ANA53145.1) are in parentheses. (**b**) The neighbor-joining tree of deduced amino acid sequences of Lac from *G. boninense* PER71 and other fungal Lacs. Numbers at the branches show bootstrap support of 1000 replicates. The tree is drawn to scale, with branch lengths having the same units to evolutionary distance denoted by the horizontal bar (0.05 amino acid substitution per site). Lac sequences obtained from this study are labeled with black circles. Abbreviations of Lacs from: *Aspergillus niger* (AnLac, GAQ36284.1); *Curvularia lunata* (ClLac, AFH53063.1); *Ganoderma fornicatum* (GfLac, ABK59827.1); *Ganoderma lucidum* (GlLac, ACR24357.1); *Ganoderma sinense* (GsLac, PIL29720.1); *Ganoderma tsugae* (GtLac, AKP24382.1); *Ganoderma weberianum* (GwLac, ANA53145.1); *Neurospora crassa* (NcLac, AAA33591.1); *Phanerochaete flavidoalba* (PfLac, ABR15762.1); *Pleurotus eryngii* (PeLac, AGO64759.1); *Pleurotus ostreatus* (PoLac, AGO64760.1); *Trametes cinnabarina* (TcLac, AAC39469.1); *Trametes versicolor* (TveLac, AAC49828.1) and *Trametes villosa* (TvLac, AAB47735.2; TviLac, Q99056.2). The vertical bars indicate the *Ganoderma* Lacs.

Since *G. boninense* is a white rot fungus which can degrade lignin, it is crucial to investigate the regulation of genes encoding lignolytic enzymes that contribute to BSR. In this study, the transcript sequences for GbMnP and GbLac, that were previously found to be present in infected oil palm [15], were successfully isolated by RACE-PCR. These sequences shared high identities with those from *Ganoderma applanatum* or *G. lucidum* with an *E*-value of 0. The signal peptide and cleavage site were predicted in the predicted amino acid sequence of these GbMnPs and GbLacs (Figure 1a and Figure 2a). Since MnP and Lac are extracellular enzymes [7,11], the signal peptide may facilitate the secretion of these lignin degrading enzymes. The distal histidine, distal arginine (as acid-base catalyst), proximal histidine, H-bonded aspartate (which lowers redox potential of heme iron) and manganese binding sites (for manganese binding which enhances MnP activity) [7] were conserved in GbMnPs (Figure 1a). The GbMnPs were clustered together with sequences from other *Ganoderma* spp. in a separate group from the fungal LiP clade (Figure 1b). Four conserved copper-binding domains where copper binds to enhance Lac activity were identified in GbLacs [11]. GbLacs were clustered together with Lacs from other *Ganoderma* spp. (Figure 2b). GbLac_U36023, which has a longer C-terminus, was found to be distinct from the other two GbLacs.

### 3.2. Enzyme Activities of MnP and Lac in G. boninense Treated with Different Nitrogen Sources, Phytohormones and H_2_O_2_

**Figure 3:**
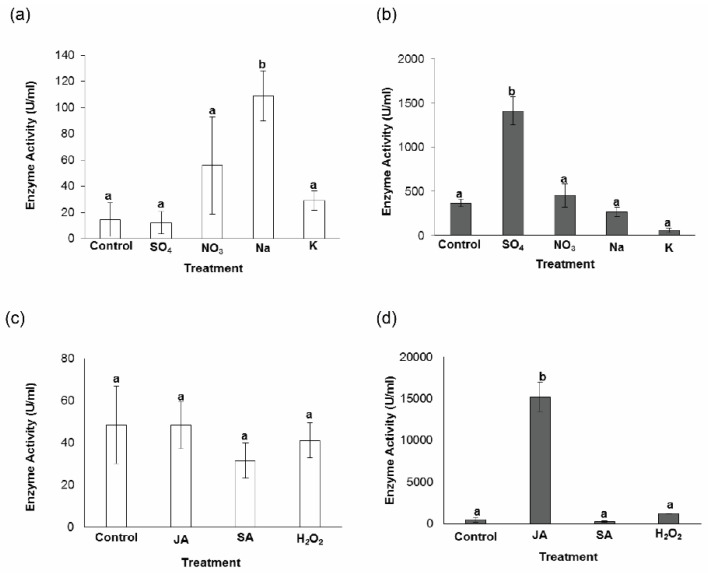
MnP and Lac enzyme activity in *Ganoderma boninense* samples in response to different nitrogen sources (**a**,**b**), phytohormones and hydrogen peroxide (**c**,**d**). (**a**,**c**) MnP activities; (**b**,**d**) Lac activities. Ammonium sulphate (SO_4_); ammonium nitrate (NO_3_); sodium nitrate (Na); potassium nitrate (K); jasmonic acid (JA); salicylic acid (SA) and hydrogen peroxide (H_2_O_2_). Differences between the means were analyzed using analysis of variance (ANOVA). Significant differences among mean values (at *p* < 0.05) were estimated using Duncan’s multiple range test as indicated by different letters.

**Figure 4:**
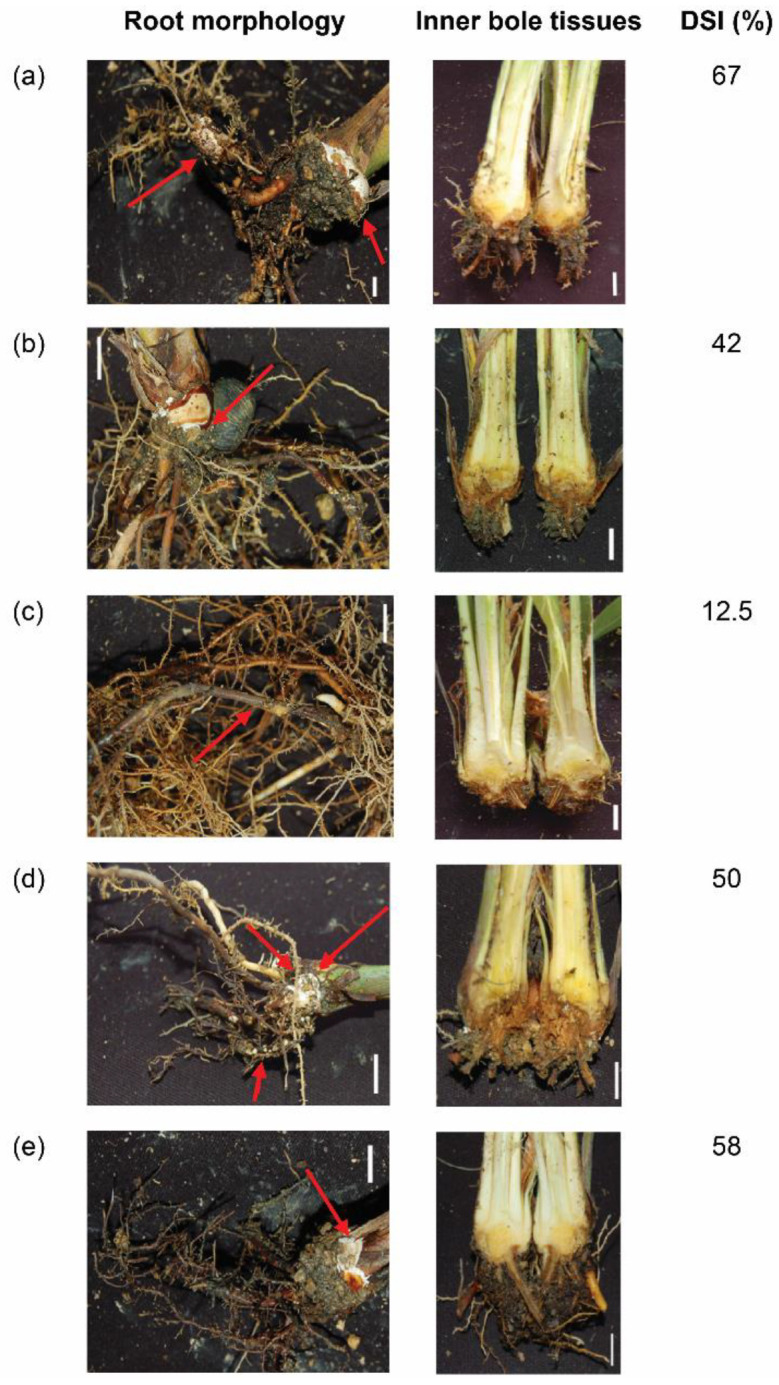
Root morphology and longitudinal section of bole tissues of *Ganoderma*-infected oil palm seedlings treated with different nitrogen sources and inoculated at 24 weeks post infection (wpi). (**a**) Without additional nitrogen source; (**b**) ammonium sulphate; (**c**) ammonium nitrate; (**d**) sodium nitrate and (**e**) potassium nitrate. Red arrow shows the formation of white mycelia or basidiocarp, and lesion sites on the roots. DSI indicates disease severity index. Scale bar represents 1 cm.

Since the enzyme activities of the treated fungal culture may reflect the effects of a treatment on the levels of MnP and Lac, we also measured the MnP and Lac activities in the *G. boninense* in vitro cultures (Figure 3) used for transcript profiling of *GbMnP*s and *GbLac*s. However, the measured enzyme activity in each culture may be contributed by more than one enzyme isoform and cannot be separated individually and might thus not necessarily agree with the transcript abundance of individual genes. In addition, we also investigated effects of nitrogen sources on the DSI of oil palm (Figure 4) caused by *G. boninense*. Meanwhile, the effects of JA and SA on the DSI of oil palm caused by *G. boninense* was inferred from the results of another in-house study which showed that the infected oil palm seedlings treated with JA and SA had no significant difference in disease symptoms compared with the untreated oil palm seedlings (unpublished data).

Figure 3 shows the enzyme activities of MnP and Lac in *G. boninense* treated with different nitrogen sources, phytohormones and H_2_O_2_. Although the mean values of MnP enzyme activity were increased by sodium nitrate, potassium nitrate and ammonium nitrate in *G. boninense* at 6 dpi (Figure 3a), only the increase of the mean value of MnP activity in *G. boninense* treated with sodium nitrate was statistically significant (*p* < 0.05). Treatment with JA, SA and H_2_O_2_ did not cause any significant changes to the mean values of MnP enzyme activity of treated *G. boninense* at 4 dpi (Figure 3c). Only treatments with ammonium sulphate and JA elevated the mean values of Lac enzyme activity in *G. boninense* (Figure 3b,d). Our results demonstrated that the enzyme activity of MnP from *G. boninense* was elevated by different nitrogen sources, whereby sodium nitrate had significant effect on the enzyme activity of MnP while ammonium sulphate caused significant increase to the enzyme activity of Lac.

Figure 4a shows that the untreated oil palm seedlings have the highest DSI (67%) compared to infected oil palms treated with different nitrogen sources, respectively. Our findings concurred with previous findings that reported that low nitrogen content may enhance delignification while high nitrogen concentrations inhibit lignin degradation [17]. We also found that infected oil palm seedlings treated with ammonium nitrate had a lower DSI compared to those in other treatments and untreated oil palm seedlings (Figure 4) although ammonium sulphate was shown to be able to increase Lac enzyme activity in the in vitro culture of *G. boninense*. Despite sodium nitrate being shown to be able to increase MnP enzyme activity in the in vitro culture of *G. boninense*, the oil palm seedlings treated with sodium nitrate have a lower DSI (i.e., 50%) compared to the untreated oil palm seedlings. Our findings suggested that the interactions of oil palm seedlings and *G. boninense* may affect the enzyme activity of lignolytic enzymes, and the outcomes may differ from those inferred from in vitro cultures that did not interact with the plant host. The expression of GbMnPs and GbLacs in the infected oil palm seedlings treated with different nitrogen sources warrants further analysis in the future.

Although the production of lignolytic enzymes from white-rot basidiomycetes was reported to be suppressed upon application of nitrogen fertilizer [16], we found that only the mean value of Lac enzyme activity in potassium nitrate-treated *G. boninense* was lower than that of the untreated *G. boninense* but the difference was non-significant (Figure 3b). Although *G. lucidum* grown in high-nitrogen was demonstrated to produce a higher Lac level in shaken cultures than that grown in low-nitrogen by a previous study [19], our results showed that only 50 mM ammonium sulphate was able to increase the Lac enzyme activity in *G. boninense*. Our findings suggested that the source of nitrogen may play a role in affecting the enzyme activity of Lac being produced.

Growth suppression of *G. boninense* by 150 parts per million (ppm) (which is approximately 1 mM), or higher concentrations of SA, accompanied by mycelial growth recovery was previously documented [24]. The growth of *G. boninense* was also found to be completely inhibited on media containing 5 mmol benzoic acid (the precursor of SA) or SA, while the addition of 1 mmol SA inhibited MnP and Lac up to 72 and 64% [23]. Although we found that 1 mM SA could decrease the mean values of MnP and Lac enzyme activities in SA-treated *G. boninense*, the decreases were shown to be non-significant. Our finding suggested that 1 mM SA might be able to suppress mycelial growth or inhibit the activities of lignolytic enzymes, but had non-significant effects on the enzyme activity of both MnP and Lac in *G. boninense*.

Our current study found that *G. boninense* treated with 1 mM JA had increased Lac enzyme activity but not MnP enzyme activity. Previously, 254 μM MeJA was found to be able to induce ganoderic acid biosynthesis in *G. lucidum* [41] and the expression of genes involved in ganoderic acid biosynthesis in response to 50–200 μM MeJA was profiled in *G. lucidum* [42]. Although the amounts of JA at millimolar concentrations were found to be effective against *Fusarium* wilt in faba bean [43] and plant brownhopper in rice [44], 50–200 ppm JA was reported to improve the growth of *G. boninense* [24]. Our findings indicated that JA may have different effects to the growth of *G. boninense* and lignolytic enzyme activities. Since lignolytic enzymes are involved in lignin degradation, we expected the hemibiotrophic fungus to have a higher activity of these enzymes at its necrotrophic phase, possibly coinciding with the JA-dependent defense in the plant host. Although we showed a higher enzyme activity of Lac in *G. boninense* in vitro culture treated with JA, it is unknown whether endogenous plant JA, which mediates the wound-induced defense response [22], could also elevate a higher Lac enzyme activity in *G. boninense*.

The enzyme activities of both MnP and Lac in *G. boninense* treated with at 1 mM H_2_O_2_, were not significantly different from that of the untreated culture. The optimum amount of H_2_O_2_ to be applied to white rot fungi to induce lignolytic activities is not known. However, 6–8 mM H_2_O_2_ were reported to be sufficient to induce the hypersensitive response in soybean suspension cultures [45] while 0.67–60 μmol H_2_O_2_ (gFW)**^−^**^1^ was recorded for leaf tissues from different plants [46,47] (whereby 60 μmol H_2_O_2_ (gFW)^−1^ is close to 100 mM on tissue water basis [46]). Although a previous study [48] had indicated that a much lower amount of H_2_O_2_ was required to suppress fungal development and suggested that lower amounts of H_2_O_2_ and possibly phytohormones could be of biological significance, higher amounts of H_2_O_2_ in millimolar concentration were found to stimulate MnP activity in white rot fungi monocultures [26]. The effects of different concentrations of SA, JA and H_2_O_2_ on the enzyme activity of MnP and Lac by *G. boninense* could be investigated in future.

### 3.3. Transcript Abundance of MnPs and Lacs in G. boninense Treated with Different Nitrogen Sources, Phytohormones and H_2_O_2_

**Figure 5:**
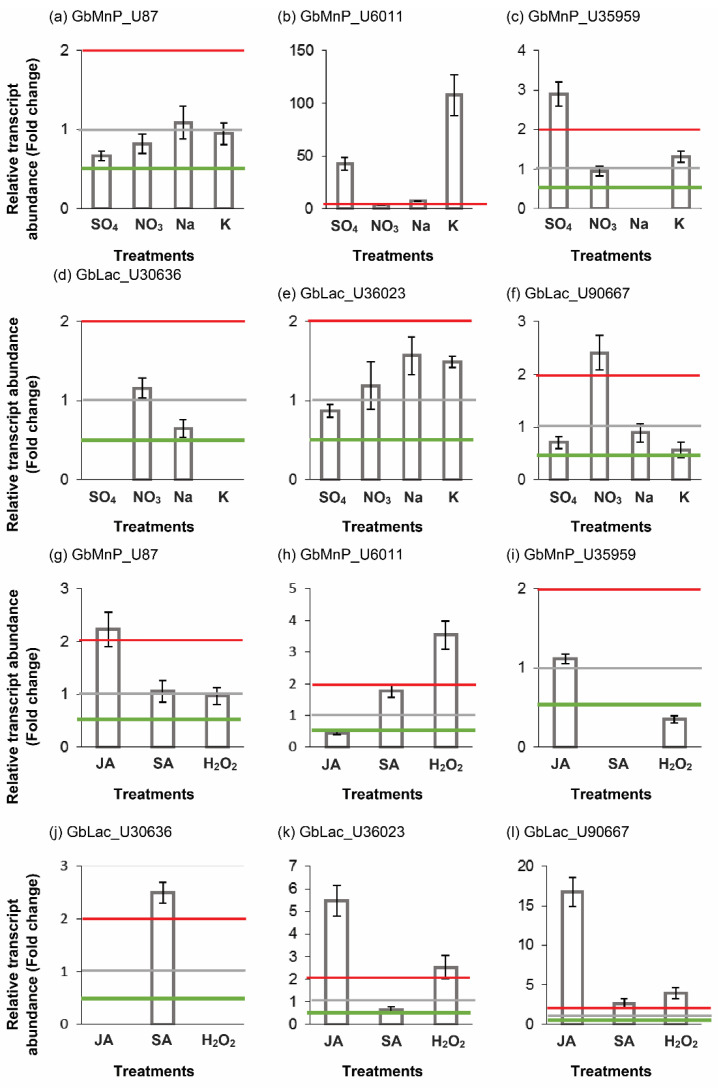
Relative expression of GbMnPs and GbLacs in *G. boninense* in response to different nitrogen sources (**a**–**f**), phytohormones and hydrogen peroxide (**g**–**l**). Ammonium sulphate (SO_4_); ammonium nitrate (NO_3_); sodium nitrate (Na); potassium nitrate (K); jasmonic acid (JA); salicylic acid (SA); hydrogen peroxide (H_2_O_2_). Red dotted lines indicate up-regulation. Green lines indicate 2-fold down-regulation. Grey lines are the normalized expression levels for untreated *G. boninense* that were grown in either Czapek–Dox broth (control for nitrogen treatments) or PDB (control for JA, SA and hydrogen peroxide treatments).

Since *G. boninense* possesses MnPs and Lacs that may have different functions and are regulated by different conditions, the relative transcript abundance of GbMnPs and GbLacs in response to different nitrogen sources, phytohormones and H_2_O_2_ was measured in this study. Of the three GbMnPs, the transcript abundance of GbMnP_U6011 showed significant changes (>2-fold) in *G. boninense* treated with different nitrogen sources (i.e., 108-fold in potassium nitrate, 43-fold in ammonium sulphate, 7.36-fold in sodium nitrate and 4.09-fold in ammonium nitrate; Figure 5b); and *GbMnP_U35959* showed an up-regulation of 2.90-fold in *G. boninense* grown in medium with ammonium sulphate in relative to that in *G. boninense* grown in Czapek–Dox broth (Figure 5c). *GbMnP_U35959* was not expressed in *G. boninense* grown in Czapek–Dox broth with sodium nitrate (Figure 5c). As for GbLacs, the transcription of GbLac_U30636 was suppressed in Czapek–Dox broth with ammonium sulphate and potassium nitrate, respectively; (Figure 5d) while the transcript abundance of GbLac_U90667 showed an increase of 2.41-fold in *G. boninense* grown in Czapek–Dox broth with ammonium nitrate in relation to that in *G. boninense* grown in Czapek–Dox broth (Figure 5f). We found that the enzyme activities of MnP and Lac in *G. boninense* did not necessary reflect the expression of individual genes encoding GbMnPs and GbLacs in *G. boninense* because the enzyme activities were contributed by all MnP and Lac enzyme isoforms in the samples while the real-time PCR analysis only measured the transcript abundance of individual genes. In addition, enzyme activities could also be affected by protein abundance that was regulated at post-transcriptional, translational and post-translational levels. Although the *G. boninense* in vitro cultures treated with sodium nitrate had a higher MnP activity, the gene expression experiments showed that none of the *GbMnP* genes analyzed was up-regulated by sodium nitrate. Similarly, none of the *GbLac* genes analyzed was up-regulated by ammonium sulphate although the treated *G. boninense* in vitro culture had a higher Lac activity. In fact, sodium nitrate and ammonium sulphate suppressed the transcription of *GbMnP_U35959* (Figure 5c) and *GbLac_U30636* (Figure 5d), respectively.

JA treatment of *G. boninense* increased the transcript abundance of GbMnP_U87 by 2.23-fold but SA and H_2_O_2_ treatments of *G. boninense* did not cause any significant changes in its gene expression compared to that of *G. boninense* grown in PDB (Figure 5g). The JA level is normally elevated in wounded host plants due to physical injury or damages inflicted by herbivores, insects and necrotrophic pathogens [22]. The expression of GbMnP_U6011 was up-regulated 3.54-fold in *G*. *boninense* treated with H_2_O_2_ but the transcript abundance of GbMnP_U6011 in *G. boninense* treated by JA was only 0.45-fold of that in PDB (Figure 5h). *GbMnP_U35959* was suppressed in *G. boninense* treated with SA while its transcript abundance in *G. boninense* treated by H_2_O_2_ was 0.35-fold of that in *G. boninense* grown in PDB (Figure 5i). Our findings showed that the transcription of GbLac_U30636 was suppressed in *G. boninense* treated with JA and H_2_O_2_ while its transcript abundance in *G. boninense* treated with SA was increased by 2.5-fold (Figure 4j). Meanwhile, the transcript level of GbLac_U36023 was increased 5.59-fold and 2.55-fold in *G. boninense* by JA and H_2_O_2_, respectively; but was reduced significantly by SA (Figure 5k). GbLac_U90667 was up-regulated 16.74-fold in *G. boninense* by JA, 2.64-fold by SA and 3.93-fold by H_2_O_2_ (Figure 5l). Coinciding with the elevation of Lac enzyme activity by JA, the gene expression of *GbLac_U36023* and *GbLac_U90667* was also up-regulated by JA. These genes may possibly encode for two of the Lac enzyme isoforms that contributed to the increase of Lac enzyme activity in JA-treated *G. boninense*.

Since *G. boninense* is a hemibiotrophic pathogen which is biotrophic at the initial stage and turns necrotrophic at a later infection stage, the endogenous SA and JA levels in the host plants may fluctuate in response to the feeding modes or infection of the pathogen. It is logical that different *GbMnPs* and *GbLacs* are present in *G. boninense* and each of them was differentially expressed in *G. boninense* in response to JA and SA in the host plants and functional at different infection stages. Although SA and JA are involved in plant defense against biotrophic and necrotrophic pathogens, respectively; while reactive oxygen species (including H_2_O_2_) are involved in both plant signaling and defense [22], it is unknown whether plant endogenous SA and JA could regulate the gene expression of *GbMnP* and *GbLac* genes as in in vitro cultures.

**Figure 6:**
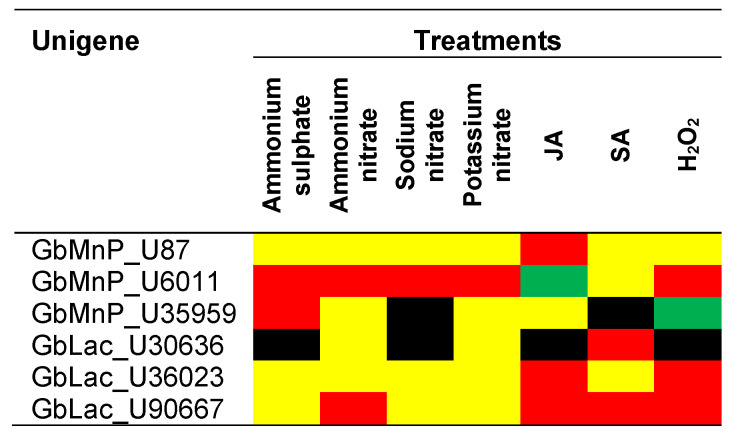
Summary of transcription regulation of genes encoding *GbMnP* and *GbLac.* Red, up-regulation ≥2-fold; green, down-regulation ≥2-fold; yellow not differentially expressed and black, suppressed.

The gene expression profile demonstrated that *GbMnP_U6011*, *GbLac_U36023* and *GbLac_U90667* was upregulated by H_2_O_2_ while the expression of *GbMnP_U35959* and *GbLac_U30636* was down-regulated and suppressed by H_2_O_2_, respectively; suggesting that individual *GbMnPs* and *GbLacs* may respond to H_2_O_2_ differently although H_2_O_2_ is required for the catalytic activities of both enzymes [28,30]. Despite the up- and down-regulation of these genes by H_2_O_2_, the enzyme activities of MnP and Lac were not significantly increased or decreased by H_2_O_2._ It is also noteworthy that the response of these *GbMnP* and *GbLac* genes to H_2_O_2_ was not always in line with that of JA or SA. Since H_2_O_2_ is a common mediator of both abiotic and biotic stresses, and can be degraded by plant enzymes including peroxidases and catalases [49], the effects of H_2_O_2_ on the gene regulation of *GbMnP*s and *GbLac*s in oil palm-*G. boninense* interaction warrant further investigation. In addition, the interactions of H_2_O_2_ with various phytohormones [50] may further complicate the gene expression profiles of these genes. The transcription regulation of these genes is summarized in Figure 6.

## 4. Conclusions

A total of six GbMnP and GbLac transcripts were successfully cloned in this study. Of the three *GbMnP* genes, *GbMnP_U6011* was up-regulated by all nitrogen sources examined and H_2_O_2_ but was down-regulated by JA. The expression of *GbMnP_U87* was only up-regulated by JA while *GbMnP_35959* was up-regulated by ammonium sulphate but suppressed by sodium nitrate and down-regulated by H_2_O_2_. Of the three *GbLac* genes analyzed, *GbLac_U90667* was up-regulated by ammonium nitrate, JA, SA and H_2_O_2_; *GbLac_U36023* was up-regulated by JA and H_2_O_2_ while *GbLac_U30636* was up-regulated by SA but suppressed by ammonium sulphate, sodium nitrate, JA and H_2_O_2_. The transcription regulation of individual *GbMnPs* and *GbLacs* may vary according to the nitrogen sources applied rather than the overall nitrogen status. Although in vitro cultures of *G. boninense* exhibited differential expression of individual *GbMnPs* and *GbLacs* in response to JA, SA and H_2_O_2_; the transcription regulation of individual *GbMnP* and *GbLac* genes and the enzyme activity of MnP and Lac enzyme isoforms in *G. boninense* interacting with its plant host warrant further investigation to verify their involvement at different infection phases of *Ganoderma*. Since the gene expression of individual genes and the total enzyme activities of MnP and Lac enzyme isoforms in the same fungal culture did not always agree with each other, enzyme assays of individual enzyme isoforms are necessary to verify their contributions to lignin degradation and BSR in oil palm. The cloning and analysis of GbMnP and GbLac transcripts in this study pave the way for recombinant protein production and enzyme assays of individual enzyme isoforms, as an alternative to purification of fungal enzymes. By identifying the main contributors of the lignolytic activities that cause BSR in oil palm, and through in-depth understanding of the regulation of these lignolytic enzymes, the formulation of nitrogen fertilizers can be modified and treatments with phytohormones and phytohormone/H_2_O_2_ inhibitors can be devised to delay or control BSR in oil palms.

## Supplementary Materials

The following are available online at https://www.mdpi.com/2073-4425/11/11/1263/s1, Table S1: Disease severity scores based on external signs and symptoms of oil palm seedlings treated with *G. boninense*; Table S2: List of primers used for cloning; Table S3: Primers (forward and reverse) for quantitative real-time PCR.

## References

[1] Turner P.D. (1981). Oil Palm Diseases and Disorders.

[2] Janusz G., Kucharzyk K.H., Pawlik A., Staszczak M., Paszczynski A.J. (2013). Fungal laccase, manganese peroxidase and lignin peroxidase: Gene expression and regulation. Enzyme Microb. Technol..

[3] Rivera-Hoyos C.M., Morales-Álvarez E.D., Poveda-Cuevas S.A., Reyes-Guzmán E.A., Poutou-Piñales R.A., Reyes-Montaño E.A., Pedroza-Rodríguez A.M., Rodríguez-Vázquez R., Cardozo-Bernal Á.M. (2015). Computational analysis and low-scale constitutive expression of laccases synthetic genes GlLCC1 from *Ganoderma lucidum* and POXA 1B from *Pleurotus ostreatus* in *Pichia pastoris*. PLoS ONE.

[4] Martínez A.T. (2002). Molecular biology and structure-function of lignin-degrading heme peroxidases. Enzyme Microb. Technol..

[5] Bourbonnais R., Paice M.G. (1990). Oxidation of non-phenolic substrates: An expanded role for laccase in lignin biodegradation. FEBS Lett..

[6] Eggert C., Temp U., Eriksson K.E.L. (1996). The ligninolytic system of the white rot fungus *Pycnoporus cinnabarinus*: Purification and characterization of the laccase. Appl. Environ. Microbiol..

[7] Giardina P., Faraco V., Pezzella C., Pisutelli A., Vanhulle S., Sannnia G. (2010). Laccases: A never-ending story. Cell Mol. Life Sci..

[8] Ko E.-M., Leem Y.-E., Choi H.T. (2001). Purification and characterization of laccase isozymes from the white-rot basidiomycete *Ganoderma lucidum*. Appl. Microbiol. Biotechnol..

[9] Teerapatsakul C., Abe N., Bucke C., Chitradon L. (2007). Novel laccases of *Ganoderma* sp. KU-Alk4, regulated by different glucose concentration in alkaline media. World J. Microbiol. Biotechnol..

[10] Kumar A., Kant K., Kumar P., Ramchiary N. (2015). Laccase isozymes from *Ganoderma lucidum* MDU-7: Isolation, characterization, catalytic properties and differential role during oxidative stress. J. Mol. Catal. Enzymat..

[11] Xu H., Guo M.Y., Gao Y.H., Bai X.H., Zhou X.W. (2017). Expression and characteristics of manganese peroxidase from *Ganoderma lucidum* in *Pichia pastoris* and its application in the degradation of four dyes and phenol. BMC Biotechnol..

[12] Zhuo R., Ma L., Fan F., Gong Y., Wan X., Jiang M., Zhang X., Yang Y. (2011). Decolorization of different dyes by a newly isolated white-rot fungi strain *Ganoderma* sp. En3 and cloning and functional analysis of its laccase gene. J. Hazard Mater..

[13] You L., Liu Z.M., Lin J.F., Guo L.Q., Huang X.L., Yang H.X. (2013). Molecular cloning of a laccase gene from *Ganoderma lucidum* and heterologous expression in *Pichia pastoris*. J. Basic Microbiol..

[14] Torres-Farradá G., Manzano León A.M., Rineau F., Ledo Alonso L.L., Sánchez-López M.I., Thijs S., Colpaert J., Ramos-Leal M., Guerra G., Vangronsveld J. (2017). Diversity of ligninolytic enzymes and their genes in strains of the genus *Ganoderma*: Applicable for biodegradation of xenobiotic compounds?. Front. Microbiol..

[15] Ho C.L., Tan Y.C., Yeoh K.A., Ghazali A.K., Yee W.Y., Hoh C.C. (2016). *De novo* transcriptome analyses of host-fungal interactions in oil palm (*Elaeis guineensis* Jacq.). BMC Genom..

[16] Magill A.H., Aber J.D. (1998). Long-term effects of experimental nitrogen additions on foliar litter decay and humus formation in forest ecosystems. Plant Soil.

[17] Kirk T.K., Schultz E., Connors W.J., Lorenz L.F., Zeikus J.G. (1978). Influence of culture parameters on lignin metabolism by *Phanerochaete chrysosporium*. Arch. Microbiol..

[18] Dill I., Kraepelin G. (1986). Palo Podrido: Model for extensive delignification of wood by *Ganoderma applanatum*. Appl. Environ. Microbiol..

[19] Rios S., Eyzaguirre J. (1992). Conditions for selective degradation of lignin by the fungus *Ganoderma australis*. Appl. Microbiol. Biotechnol..

[20] Mohd Tayeb D., Idris A.S., Mohd Haniff H. Reduction of *Ganoderma* infection in oil palm through balanced fertilization in peat. Proceedings of the International Palm Oil Congress.

[21] Pujianto, Achmad W.S., Dafian P., Syaiful, Suhardi, Putri A.W., Caliman J.P. Impact of mineral nutrition management on Ganoderma incidence in oil palm planted on peat soil. Proceedings of the 15th International Peat Congress.

[22] De Coninck B., Timmermans P., Vos C., Cammue B.P.A., Kazan K. (2015). What lies beneath: Belowground defense strategies in plants. Trends Plant Sci..

[23] Surendran A., Siddiqui Y., Saud H.M., Ali N.S., Manickam S. (2018). Inhibition and kinetic studies of lignin degrading enzymes of *Ganoderma boninense* by natural occurring phenolic compounds. J. Appl. Microbiol..

[24] Ong C.E., Goh Y.K., Tan S.Y., Goh Y.K., Goh K.J. (2018). A preliminary study on the effects of salicylic and jasmonic acids on *Ganoderma boninense* growth, mycelial hydrophobicity, and media pH under *in vitro* assays. Arch. Phytopath. Plant Prot..

[25] Rahamah Bivi M., Siti Noor Farhana M.D., Khairulmazmi A., Idris A., Ahmed O.H., Zamri R., Sariah M. (2012). In vitro effects of salicylic acid, calcium and copper ions on growth and sporulation of *Ganoderma boninense*. Afr. J. Biotech..

[26] Rahamah Bivi M., Paiko A.S., Khairulmazmi A., Akhtar M.S., Idris A.S. (2016). Control of basal stem rot disease in oil palm by supplementation of calcium, copper, and salicylic acid. Plant Pathol. J..

[27] Bermek H., Li K., Eriksson K.-E., Viikari L., Lantto R. (2002). Studies on inactivation and stabilization of manganese peroxidase from *Trametes versicolor*. 8th International Conference on Biotechnology in the Pulp and Paper Industry.

[28] Chan-Cupul W., Arámbula-Zúñiga C.S., Fan Z., Heredia G. (2019). Oxidative enzymes activity and hydrogen peroxide production in white-rot fungi and soil-borne micromycetes co-cultures. Ann. Microbiol..

[29] Li D., Alic M., Brown J.A., Gold M.H. (1995). Regulation of manganese peroxidase gene transcription by hydrogen peroxide, chemical stress, and molecular oxygen. Appl. Environ. Microbiol..

[30] Branden R., Malmstrom B., Vanngard T. (1971). The interaction of fungal laccase with hydrogen peroxide and the removal of fluoride from the inhibited enzyme. Eur. J. Biochem..

[31] Asther M., Lesag L., Drapron R., Corrieu G., Odier E. (1988). Phospholipid and fatty acid enrichment of Phanerochaete chrysosporium INA-12 relation to ligninase production. Appl. Microbiol. Biotechnol..

[32] Wolfenden B.S., Wilson R.L. (1982). Radical cations as reference chromogens in studies of one-electron transfer reactions: Pulse radio analysis studies of 2,2’-azinobis-(3-ethlbenzthiazoline-6-sulfonate). J. Chem. Soc. Perkin Trans..

[33] Zaiton S., Sariah M., Zainal A.M.A. (2008). Effect of endophytic bacteria on growth and suppression of *Ganoderma* infection in oil palm. Int. J. Agric. Biol..

[34] Song W., Zhou L., Yang C., Cao X., Zhang L., Liu X. (2004). Tomato *Fusarium* wilt and its chemical control strategies in a hydroponic system. Crop Prot..

[35] Wang T., Zhang N., Du L. (2005). Isolation of RNA of high quality and yield from *Ginkgo biloba* leaves. Biotechnol. Lett..

[36] Hall T.A. (1999). BioEdit: A user-friendly biological sequence alignment editor and analysis program for Windows 95/98/NT. Nucleic Acids Symp. Ser..

[37] Tamura K., Peterson D., Peterson N., Stecher G., Nei M., Kumar S. (2011). MEGA5: Molecular evolutionary genetics analysis using maximum likelihood, evolutionary distance, and maximum parsimony methods. Mol. Biol. Evol..

[38] Armenteros J.J.A., Tsirigos K.D., Sonderby C.K., Petersen T.N., Winther O., Brunak S., von Heijne G., Nielsen H. (2019). SignalP 5.0 improves signal peptide predictions using deep neural networks. Nat. Biotechnol..

[39] Lim F.H., Nor Fakhrana I., Abdul Rasid O., Idris A.S., Ahmad Parveez G.K., Ho C.L., Shaharuddin N.A. (2014). Isolation and selection of reference genes for *Ganoderma boninense* gene expression study using quantitative real-time PCR (qPCR). J. Oil Palm Res..

[40] Tan Y.C., Yeoh K.A., Wong M.Y., Ho C.L. (2013). Expression profiles of putative defence-related proteins in oil palm (*Elaeis guineensis*) colonized by *Ganoderma boninense*. J. Plant Physiol..

[41] Ren A., Qin L., Shi L., Dong X., Mu D.S., Li Y.X., Zhao M.W. (2010). Methyl jasmonate induces ganoderic acid biosynthesis in the basidiomycetous fungus *Ganoderma lucidum*. Bioresour. Technol..

[42] Ren A., Li M.-J., Shi L., Mu D.-S., Jiang A.-L., Han Q., Zhao M.-W. (2013). Profiling and quantifying differential gene transcription provide insights into ganoderic acid biosynthesis in *Ganoderma lucidum* in response to methyl jasmonate. PLoS ONE.

[43] Ahmed H.F.S., El-Arab M.M., Omar S.A. (2002). Differential effect of jasmonic acid on the defense of faba bean sgainst Fusarium Wilt: Modulation of other phytohormones and simple phenols. Int. J. Agri. Biol..

[44] Sengottayan S.-N., Kandaswamy K., Choi M.Y., Paik C.-H. (2009). Effects of jasmonic acid-induced resistance in rice on the plant brownhopper, *Nilaparvata lugens* Stål (Homoptera: Delphacidae). Pestic. Biochem. Physiol..

[45] Levine A., Tenhaken R., Dixon R.A., Lamb C.J. (1994). H_2_O_2_ from the oxidative burst orchestrates the plant hypersensitive disease resistance response. Cell.

[46] Cheeseman J.M. (2006). Hydrogen peroxide concentrations in leaves under natural conditions. J. Exp. Bot..

[47] He Y.L., Liu Y.L., Cao W.X., Huai M.F., Xu B.G., Huang B.G. (2005). Effects of salicylic acid on heat tolerance associated with antioxidant metabolism in Kentucky bluegrass. Crop Sci..

[48] Aver‘yanov A.A., Lapikova V.P., Pasechnik T.D., Kuznetsov V.V., Baker C.J. (2007). Suppression of early stages of fungus development by hydrogen peroxide at low concentrations. Plant Pathol. J..

[49] Smirnoff N., Arnaud D. (2019). Hydrogen peroxide metabolism and functions in plants. New Phytol..

[50] Saxena I., Srikanth S., Chen Z. (2016). Cross talk between H_2_O_2_ and interacting signal molecules under plant stress response. Front. Plant Sci..

