# Supplementary material for: Expression of Genes Encoding Manganese Peroxidase and Laccase of Ganoderma boninense in Response to Nitrogen Sources, Hydrogen Peroxide and Phytohormones
Source: Genes (Basel). 2020 Oct 26;11(11):1263. doi: 10.3390/genes11111263 (PMC7692562; doi:10.3390/genes11111263)
Supplement: Supplementary file 1 [file genes-11-01263-s001.pdf]

Table S1. Disease severity scores based on external signs and symptoms of oil palm seedlings treated with *G. boninense*.

Scale	Signs and symptoms
0<br>Healthy	Healthy roots without mycelia on roots surface
1<br>Mild infection	Mycelial mass on roots surface
2<br>Moderate infection	Mycelia and lesion on roots surface
3<br>Severe infection	Basidiocarp formation on roots
4<br>Very severe infection	Plant death with well-developed basidiocarp

Modified from Abdullah F, Ilias GNM, Nelson M, Mohd-Zainudin NAI, Umi Kalsom Y. Disease assessment and the efficacy of Trichoderma as a biocontrol agent of basal stem rot of oil palm. Science Putra Research Bulletin 2003;11:31-33.

Table S2. List of primers used for cloning.

Primer Name	Sequence 5' - 3'	Length (Number of nucleotides)	GC Content (%)	Tm (°C)
**For 3'- RACE-PCR**				
U87-RACE-FMNP*	GCAGCCCTTTCACCATCCTCCTCAT	25	56.0	66.0
U87-RACE-FMNP2	CATCCTCCTCATCCTCCTAATTCGCC	26	53.9	64.6
U35959-3RACE-FMNP1*	CACGACGCTCTGGCCTACTCTCCTGCA	27	63.0	70.5
U35959-3RACE-FMNP2	CGGAGGGAAAATTCGGCGGCGGAGG	25	68.0	75.3
U30636-RACE-FLAC*	CAGCCTTTCGAGTCCCTCACCTCTT	25	56.0	63.7
U30636-RACE-FLAC2	CGAGTCCCTCACCTCTTATCCTA	23	52.2	55.3
U36023-RACE-FLAC1*	TCAACTCTGCCATCCTGCGCTACTC	25	56.0	64.4
U36023-RACE-FLAC2	ACGACCAACCAGACCACATCTGTCC	25	56.0	63.9
**For 5'-RACE-PCR**				
U6011-RACE-FMNP1*	TCCGTGATACAAACAAGCCCTCGTTCC	27	51.9	67.2
U6011-RACE-FMNP2	GGATGTATGAGCGAGTCGTTCCTCCCT	27	55.6	66.0
U35959-5RACE-FMNP1*	GCCCTCCAAGAATGTTGCGCACTGCTCG	28	60.7	74.1
U35959-5RACE-FMNP2	GGTACGCCAATAATGGCTTGACGGCCGG	28	60.7	73.7
U36023-5RACE-FLAC1*	GACGTCGTTGATGAAGAACTCGGTACCG	28	53.6	67.0
U36023-5RACE-FLAC2	GCGTAGTCGACACCACCAACCTCA	24	58.3	63.9
**For full-length-PCR cloning**				
U36023-FL-FLAC (F)	CCCTCCTCAGCGATCCTACAG	21	61.9	57.4
U36023-RT-RLAC (R)	GATACAACCGCTACTAAGCAG	21	47.6	48.6
U36023-FL-FLAC (F)	CCCTCCTCAGCGATCCTACAG	21	61.9	57.4
U36023-RT-RLAC (R)	GATACAACCGCTACTAAGCAG	21	47.6	48.6

*****Primer names with (*) are for primary RACE-PCR while primer names without (*) are for nested RACE-PCR; *(F) indicates forward primer and (R) indicates reverse primer.

Table S3. Primers (forward and reverse) for quantitative real-time PCR.

Primer Name	Sequence 5'-3'	Length (bp)	GC Content (%)	Tm (°C)
U87-RT-FMNP4*	CTGTCACTTCTATCCCTGCC	20	55.0	56.4
U87-RT-RMNP4	CATCGCCCGAAATACACCTCA	21	52.4	59.5
U6011nU97010-RT-FMNP*	ACGGTGAACACTGTTGCT	18	50.0	47.7
U6011-RT-RMNP4	AAGTGGGATGTATGAGCGAGTC	21	47.6	56.6
U35959-RT-FMNP*	CGGTCTCCACTGTCTCCGGCACT	23	65.2	65.3
U35959-RT-RMNP2	CTACAATGCCCTCCAAGAATG	21	47.6	55.7
U30636-RT-FLAC*	TTCCAGACGAACAACCCG	18	55.6	53.8
U30636-RT-RLAC2	GCCACCTTCGACATCCAA	18	55.6	53.6
U36023-RT-FLAC2*	ACGAGAGCTTCTAGGCGA	18	55.6	49.6
U36023-RT-RLAC2	TAGAGAACTCGAATGCCCG	19	52.6	52.5
U90667-RT-FLAC*	TTCCAGACGAACAACCCG	18	55.6	53.8
U90667-RT-RLAC	AACTCAGAAGCCCAAAGC	18	50.0	48.9
α-tubulin-GTR 7*	GCACCGACTCTGGTGATGCT	20	60.0	57.2
α-tubulin-GTR 8	GATAGGCTATGGTCGCGAAG	20	55.0	53.8
β-tubulin-GTR 3*	GAGTTCACTGAGGCCGAGTC	20	60.0	53.3
β-tubulin-GTR 4	TGCAACACGCTTATTCTTCG	20	45.0	53.8

*Primer names with (*) are forward primers, without (*) are reverse primers. Primers of alpha and beta tubulin were obtained from Lim *et al*. (2014)
